# Predictors of acute adverse reactions to non-ionic iodinated contrast media in CT imaging: a systematic review and meta-analysis

**DOI:** 10.3389/fradi.2025.1656949

**Published:** 2025-09-19

**Authors:** Ke Liu, Xin Cheng, Yongli Zhu, Jun Long, Changsheng Li, Lijun Cui, Kang Li, Changping Mu

**Affiliations:** ^1^ Department of Nursing, North Sichuan Medical College, Nanchong, China; ^2^ Department of Radiology, Chongqing General Hospital, Chongqing University, Chongqing, China; ^3^ Department of Nursing, Affiliated Hospital of North Sichuan Medical College, Nanchong, China; ^4^ Chongqing University FuLing Hospital, Chongqing, China; ^5^ Army Characteristic Medical Center of PLA, Chongqing, China

**Keywords:** iodine, contrast media, acute adverse reactions, influencing factors, meta-analysis

## Abstract

**Background:**

Iodinated contrast media-acute adverse reactions (ICM-AARs) are frequent and clinically significant complications associated with radiological imaging. Despite investigation of their risk factors, there is no consensus, and no comprehensive synthesis has been conducted. This systematic review and meta-analysis aimed to investigate the factors influencing ICM-AARs.

**Methods:**

A systematic search for studies published in Chinese or English up to 22 July 2024 in the PubMed, Web of Science, Cochrane Library, Embase, CNKI, WanFang, CQVIP, and SinoMed databases was conducted. Studies on patients undergolng contrast-enhanced CT examinations with nonionic ICM were selected. The primary outcome measures were risk factors associated with ICM-AARs. The studies were analyzed for heterogeneity using the *Q*-test and I^2^ statistic, while publication bias was assessed using funnel plots, Egger's test, and Begg's test. Stata 17 software was used for the meta-analysis.

**Results:**

Seventeen studies were included, encompassing 2,576,446 CT-enhanced examinations. Of these, 11,621 acute adverse reactions were reported, with a mean incidence of 0.45% and a quality score of ≥7. The meta-analysis showed that female sex (OR = 1.27, 95% CI = 1.13, 1.41), age <35 years (OR = 1.77, 95% CI = 1.19, 2.64), high body mass index (OR = 1.06, 95% CI = 1.01, 1.10), type of medical visit (outpatient) (OR = 2.23, 95% CI = 1.01, 4.93), history of adverse ICM reactions (OR = 11.03, 95% CI = 2.25, 53.97), history of other allergies (OR = 3.16, 95% CI = 1.27, 7.84), history of asthma (OR = 1.75, 95% CI = 1.19, 2.57), hyperthyroldism (OR = 4.59, 95% CI = 1.65, 12.82), and type of ICM (OR = 2.27, 95% CI = 1.68, 3.06) were risk factors for ICM-AARs. Age >60 years (OR = 0.71, 95% CI = 0.53, 0.95), pre-injection medication (OR = 0.56, 95% CI = 0.39, 0.79), and hypertensive disorders (OR = 0.78, 95% CI = 0.65, 0.94) were identified as protective against ICM-AARs.

**Conclusions:**

The incidence of ICM-AARs is influenced by a variety of clinical and demographic factors. Healthcare professionals may benefit from dynamically assessing patient-specific risk factors and considering targeted preventive measures for high-risk groups, particularly in populations similar to those studied.

**Systematic Review Registration:**

https://www.crd.york.ac.uk/PROSPERO/, PROSPERO (CRD42024571470).

## 1 Introduction

Iodinated contrast media (ICM) is an important auxiliary tool in medical imaging as it can distinguish among different tissues, thereby enhancing diagnostic precision. ICMs include both ionic and non-ionic types, of which non-ionic ICMs are currently the most widely used contrast media in CT enhancement due to their greater safety (1). As of 2017, ICM has been used more than 100 million times per year worldwide (2). Although ICM is well tolerated, 0.4%‒1.3% of the population may still experience ICM-related adverse reactions (3–5), ranging from mild nausea, vomiting, and skin itching to severe anaphylactic shock and death (6, 7). ICM adverse reactions are classified according to the time of appearance into acute adverse reactions (AARs) (≤1 h) and delayed adverse reactions (>1 h) (8–10), with AARs categorized into allergic (hypersensitivity) and non-allergic (physiological) reactions; however, due to the limited data available, the present study does not provide a stratified analysis of these subtypes. AARs include nausea and vomiting, allergic symptoms, and laryngeal edema, among others. Approximately 90% of adverse reactions are AARs (11), with almost all potentially life-threatening adverse reactions occurring within 20 min of ICM injection (12). Therefore, identification of the factors influencing ICM-AARs is crucial. Various influencing factors have reported in recent years, and a comprehensive understanding of them would contribute significantly to the early recognition and treatment of ICM-AARs. Therefore, this meta-analysis aimed to synthesize current evidence on factors influencing the onset of ICM-AARs. The findings would support hypothesis generation and inform clinical strategies for identifying potentially high-risk populations. In addition, the results may help inform patient education in the risks associated with contrast media, optimize risk stratification protocols before contrast administration, and guide institutional policies on the selection of contrast media.

## 2 Materials and methods

### 2.1 Study registration

The research followed the Priority Reporting Items for Systematic Reviews and Meta-Analyses (PRISMA) reporting standards (13). The protocol has been registered with the International Prospective Register of Systematic Reviews and Meta-Analyses (PROSPERO) [CRD42024571470].

### 2.2 Literature search

The PubMed, Web of Science, Cochrane Library, Embase, CNKI, WanFang Database, CQVIP, and SinoMed databases were comprehensively searched for relevant studies. The search period spanned from database inception to July 2024. Searches utilized both medical subject headings (MeSH) and free-text terms, including “contrast media”, “iodinated contrast media”, “drug-related side effects”, “adverse reactions”, “acute adverse reactions”, and “risk factors”. Details of the search strategies used for the different databases are shown in Supplementary Table S1A.

### 2.3 Selection criteria

The inclusion criteria were as follows: (1) The study type was a cohort study, case-control study, or cross-sectional study; (2) The study population consisted of patients with CT-enhanced non-ionic ICM-AARs; (3) The outcome measure factors affecting ICM-AARs, and the study data could be extracted as odds ratios (ORs), hazard ratios (HRs), risk differences (RDs), and 95% confidence intervals (CIs); (4) The language of the publication was either Chinese or English.

The exclusion criteria were as follows: (1) Duplicate studies; (2) Review articles, Conference abstracts, meta-analyses, and animal experiments; (3) Unavailability of full text or incomplete data; (4) Low-quality studies.

### 2.4 Data extraction

After the literature screening, two researchers (Liu, K and Zhu, YY) independently extracted the information from the articles. The extracted data primarily included the following variables: author, time of publication, country, number of CT examinations, number of AARs, incidence, study design, and influencing factors. In cases of disagreement, discussions were held and resolved with a third investigator (Long, J).

### 2.5 Risk of bias assessment

Two researchers (Liu, K and Zhu, YY) independently evaluated the quality of the included studies for risk of bias, and any disagreement during the evaluation process was resolved through discussion or consultation with a third researcher (Long, J). Case-control studies and cohort studies were scored using the Newcastle Ottawa Scale (NOS) (14) with a total score of 9, where scores of 0‒3 indicated low quality, 4‒6 indicated moderate quality, and 7‒9 indicated high quality. Cross-sectional studies were evaluated using the quality assessment criteria recommended by the Agency for Healthcare Research and Quality (AHRQ) (15), with a total score of 11, where 0‒3 indicated low quality, 4‒7 represented moderate quality, and ≥8 indicated high quality. Studies with scores ≥6 were classified as high-quality studies. To ensure the quality of the articles, studies with scores <6 were excluded from the analysis.

### 2.6 Statistical analysis

Stata 17 software was used for data analysis, combining the ORs and 95% CIs from the multifactorial analyses, with a significance level of *α* = 0.05, giving preference to multifactorial-adjusted data. The ORs were converted into log(OR) and their standard errors (SE). The combined OR estimates were calculated using both fixed and random effects models. Judgments were based on the results of the heterogeneity test (*Q*-test method) and the I^2^ statistic (16); values of *P* > 0.1 and I^2^ < 50% represented acceptable heterogeneity between studies, and a fixed-effects model was used, while values of *P* ≤ 0.1 and I^2^ ≥ 50% indicated significant heterogeneity between the studies. Sources of heterogeneity were explored through subgroup analyses (17). If no source of heterogeneity was identified, a random-effects model was used. Sensitivity analyses were performed using a case-by-case exclusion method. When the number of articles included in a single outcome was ≥10, a funnel plot was drawn (18), and Egger's and Begg's tests were used to assess publication bias.

## 3 Results

### 3.1 Study identification

**Figure 1:**
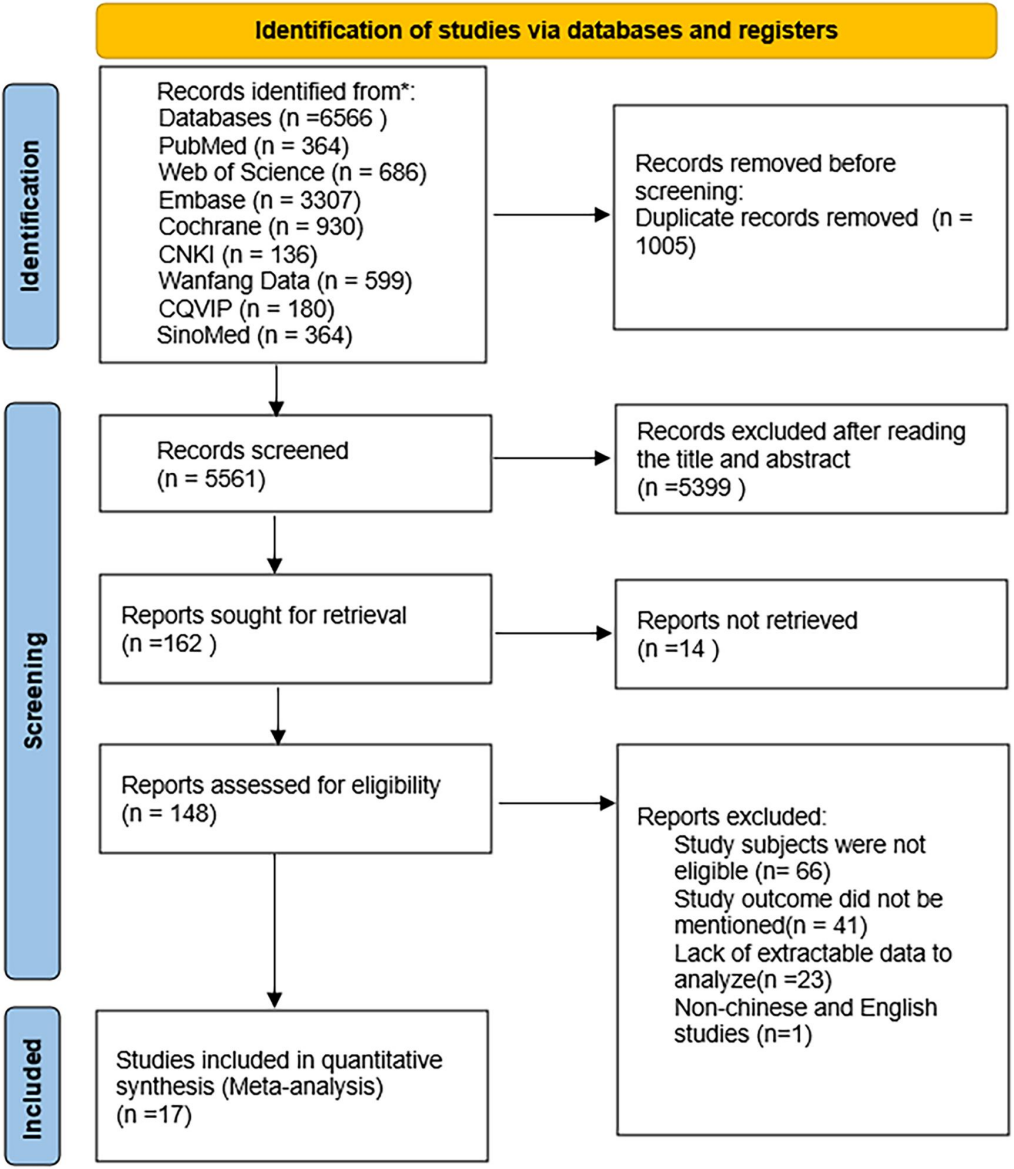
Flowchart of literature screening.

The database search yielded 6,566 potentially eligible articles, including 5,287 in English and 1,279 in Chinese. Duplicate studies totaled 1,005 articles and were excluded. A further 5,399 articles were excluded as their titles and abstracts did not meet the inclusion and exclusion criteria. After reviewing the full texts of 164 articles, 17 were finally included (19–35), with 7 in Chinese (19–25) and 10 in English (26–35). The literature screening process is illustrated in Figure 1.

### 3.2 Study characteristics and risk of bias assessment

Of the 17 studies ultimately included, 4 were cohort studies, 10 were case-control studies, and 3 were cross-sectional studies. The included studies were from China (*n* = 8), the USA (*n* = 1), Japan (*n* = 1), South Korea (*n* = 5), and Australia (*n* = 1). A total of 25,764,46 CT enhancement examinations were described in the studies, among which 11,621 cases of ICM-AARs occurred, with a mean incidence rate of 0.45%. Thirty-eight influencing factors were described. The NOS and AHRQ scores ranged from 6 to 8, indicating a low risk of bias. The fundamental characteristics and quality assessment results are presented in Table 1. The details of the NOS and AHRQ scores are shown in Supplementary Table S1B.

**Table 1:** The fundamental characteristics and quality assessment results (*n* = 17).

Author	Year	Study country	Examination (number)	AARs (number)	Incidence	Study design	Influential factors	Quality score
Yang et al.	2023	China	5,885	160	2.72%	Cross-sectional	Item 1, 2, 4, 5, 13, 17, 25, 26, 28–34	7
Xu et al.	2023	China	800	80	10%	Cohort	Item 6, 7, 9, 12	7
Qiu et al.	2023	China	332,683	931	0.28%	Case-control	Item 1, 2, 5, 10–12, 14, 16, 38	8
Gao et al.	2023	China	1,260	18	1.43%	Case-control	Item 10, 12, 14, 24, 25, 28	8
Ding et al.	2023	China	26,871	89	0.33%	Case-control	Item 2, 3, 12, 15, 28, 35, 36	8
Lin et al.	2022	China	162,073	242	0.15%	Case-control	Item 1, 2, 12, 18, 28, 30	7
Gan et al.	2020	China	55,855	38	0.07%	Case-control	Item 2, 28	8
Zeng et al.	2024	China	473,482	469	0.1%	Case-control	Item 16, 21–23, 38	8
McDonald et al.	2023	USA	359,977	1,829	0.51%	Case-control	Item 1, 2, 4, 5, 8, 10–12, 14, 26, 28, 37	7
Liu et al.	2023	China	271,165	920	0.34%	Cohort	Item 25, 26	7
Fukushima et al.	2023	Japan	76,194	45	0.06%	Case-control	Item 1, 2, 5, 19, 28	7
Park et al.	2019	Korea	52,293	844	1.61%	Cohort	Item 1–3, 10, 13, 27, 28, 37	8
Lee et al.	2019	Korea	205,726	2,004	0.97%	Cross-sectional	Item 1, 2, 10, 12, 14	6
Cha et al.	2019	Korea	196,081	1,433	0.73%	Cross-sectional	Item 10–12, 14, 15, 20	6
Kim et al.	2017	Korea	286,087	1,969	0.69%	Case-control	Item 1–3, 28, 37	8
Yang et al.	2015	Korea	40,052	503	1.26%	Cohort	Item 1, 2, 28	7
Ho et al.	2012	Australia	29,962	47	0.16%	Case-control	Item 1, 2, 5, 38	7

Item 1: Sex; item 2: Age; item 3: Weight; item 4: Body mass index; item 5: Type of medical treatment; item 6: Educational level; item 7: Income; item 8: Race; item 9: Anxiety; item 10: History of adverse reaction to ICM; item 11: No previous allergy to ICM; item 12: History of other allergies; item 13: Pre-injection medication; item 14: History of asthma; item 15: Hyperthyroldism; item 16: Hypertensive disorders; item 17: History of chemotherapy; item 18: History of surgery; item 19: Preoperative medication; item 20: Family history; item 21: Heart disease; item 22: Hypertension plus heart disease; item 23: Diabetes; item 24: Diabetic nephropathy; item 25: Injection flow rate; item 26: Injection volume; item 27: Contrast concentration; item 28: Type of ICM; item 29: First CT enhancement; item 30: Duration of fasting prior to examination; item 31: Oral hydration; item 32: Intravenous hydration; item 33: Estimated glomerular filtration rate; item 34: Examination items: organs/vessels; item 35: Temperature; item 36: Humidity; item 37: CT examination site; item 38: Season.

### 3.3 Results of meta-analysis of factors influencing ICM-AARs

We analyzed the risk factors associated with ICM-AARs by combining the ORs from the multifactorial analyses. Seventeen studies with ≥2 influencing factors were combined, resulting in a total of 16 influencing factors. The results of the meta-analysis showed that female sex, age, high body mass index, type of medical treatment, history of adverse reaction to ICM, history of other allergies, history of asthma, hyperthyroldism, and type of ICM iodine contrast media were risk factors for ICM-AARs. In contrast, pre-injection medication and hypertension were found to be protective against ICM-AARs. There was insufficient evidence to suggest an association between other factors (e.g., body weight, previous ICM allergy-free use, injection dose, site of CT examination, and season) and ICM-AARs. The details are provided in Table 2.

**Table 2:** Analysis of factors influencing ICM-AARs.

No.	Category	Influencing factor	Number of included studies	Heterogeneity		Effect model	Meta-analysis results	
				I^2^ (%)	*p*-value		Odds ratio	95% CI
1	Socio-demographic factors	Female	10	68.9	<0.001	Random	1.27	[1.13, 1.41]
2		Age	12	94.9	<0.001	Random	1.07	[1.03, 1.11]
3		Weight	3	92.1	<0.001	Random	1.01	[1.00, 1.02]
4		Body mass index	2	47.1	0.129	Fixed	1.06	[1.01, 1.10]
5		Type of medical treatment	5	96.4	<0.001	Random	2.23	[1.01, 4.93]
6	Disease-related factors	History of adverse reactions to ICM	6	99.3	<0.001	Random	11.03	[2.25, 53.97]
7		No previous allergy to ICM	3	95.7	<0.001	Random	0.76	[0.47, 1.21]
8		History of other allergies	8	99.1	<0.001	Random	3.16	[1.27, 7.84]
9		Preinjection medication	2	7.1	0.341	Fixed	0.56	[0.39, 0.79]
10		History of asthma	5	86.9	<0.001	Random	1.75	[1.19, 2.57]
11		Hyperthyroldism	2	0	0.435	Fixed	4.59	[1.65, 12.82]
12		Hypertensive disorders	2	0	0.688	Fixed	0.78	[0.65, 0.94]
13	ICM-related factors	Injection volume	2	81.2	0.021	Random	1.12	[0.88, 1.43]
14		Type of ICM	8	89.7	<0.001	Random	2.27	[1.68, 3.06]
15	Other factors	CT examination site	3	98.9	<0.001	Random	0.88	[0.35, 2.22]
16		Season	3	86.5	<0.001	Random	1.08	[0.81, 1.45]

**Figure 2:**
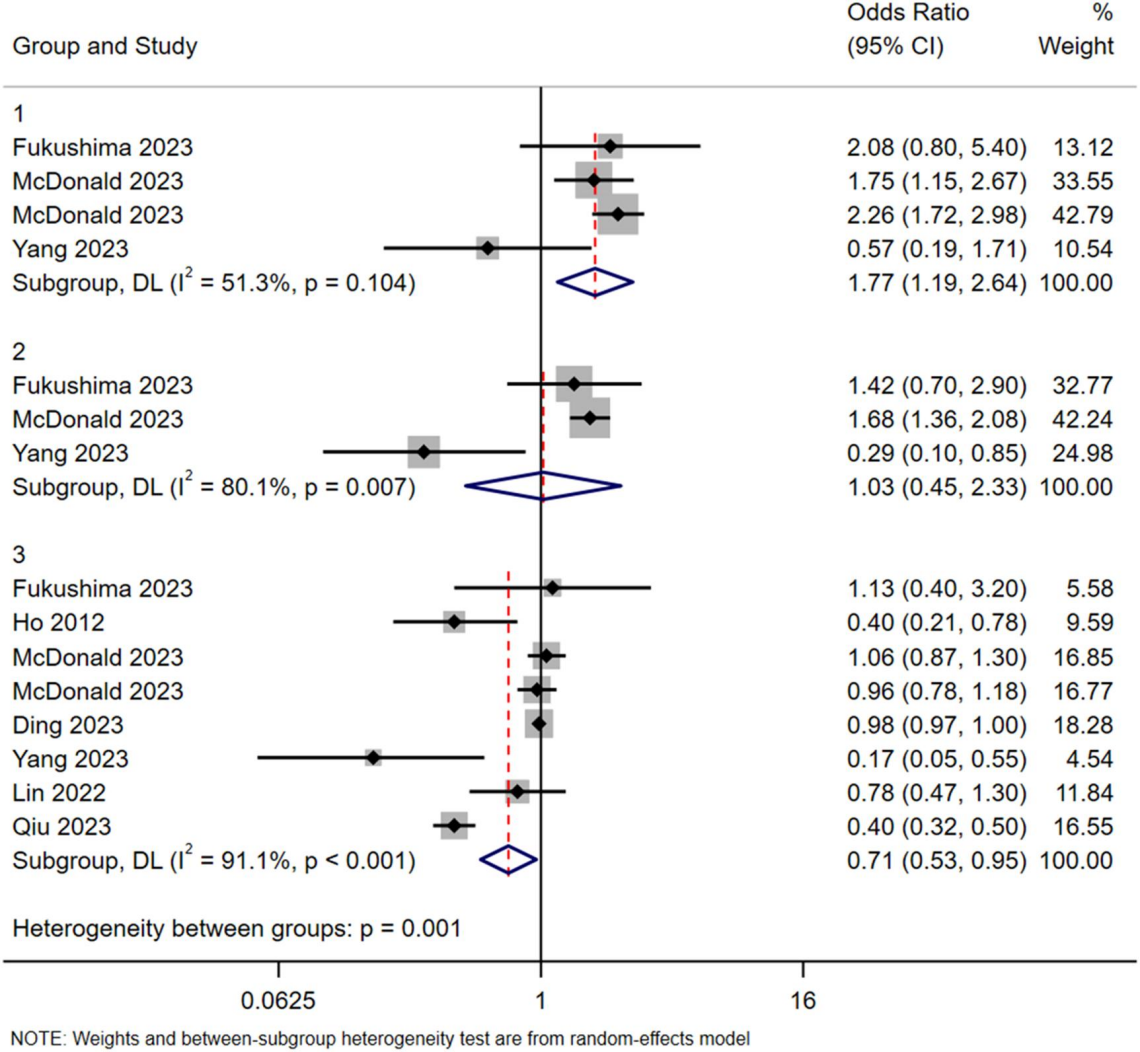
Subgroup analysis in terms of age. Group 1: <35 years old; Group 2: 35–60 years old; Group 3: >60 years old.

In addition, subgroup analyses based on age and type of contrast agent were conducted. In terms of age, participants were divided into a young group (<35 years), a middle-aged group (35–60 years), and an older group (>60 years). The results suggested a lower reported incidence of ICM-AARs in the older group, although this finding should be interpreted cautiously given the potential influence of clinical and reporting biases (Figure 2).

**Figure 3:**
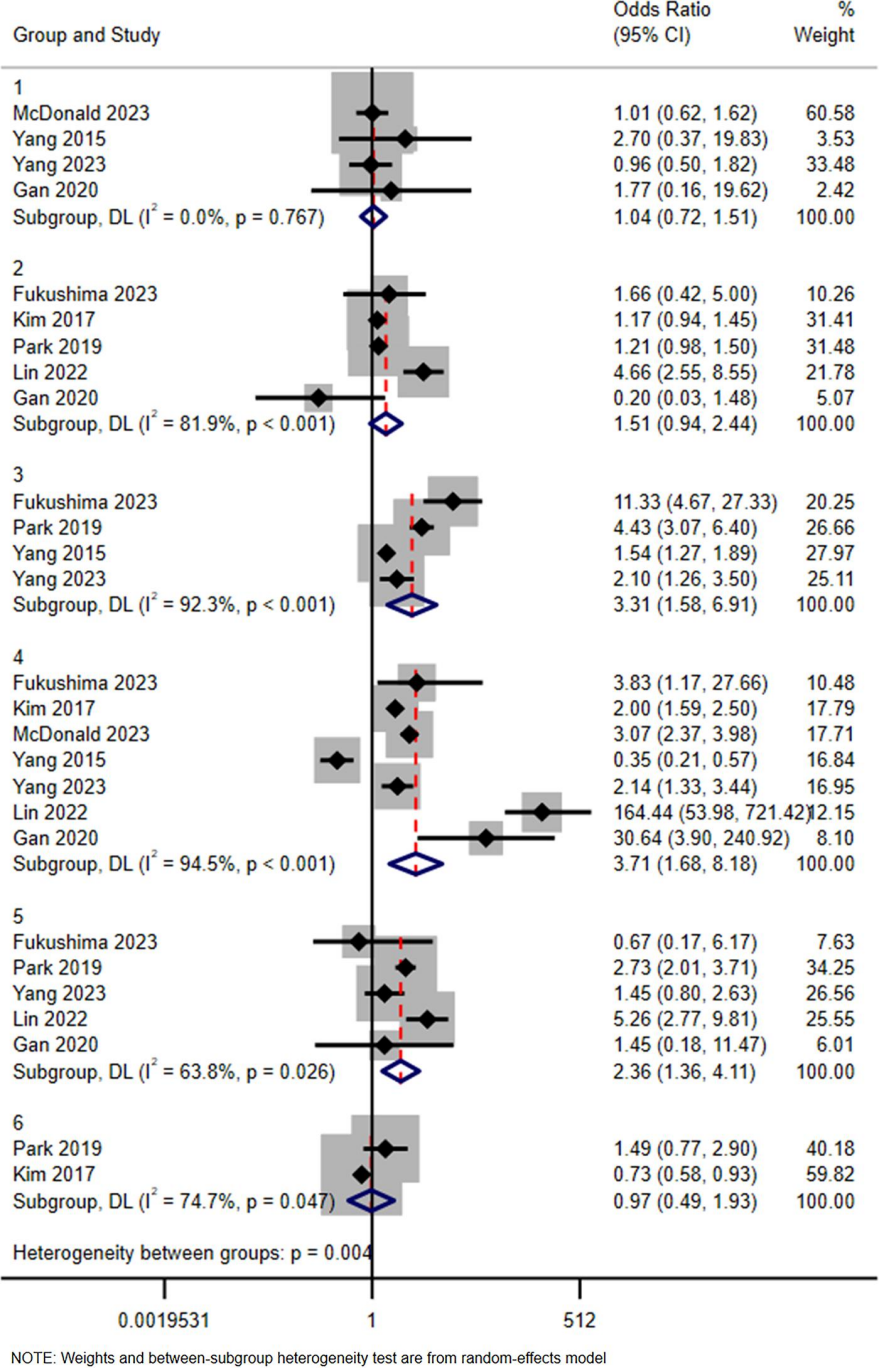
Subgroup analysis in terms of type of iodine contrast media (iohexol represents the reference). Group 1: Iodixanol; Group 2: Iopamidol; Group 3: Iomeprol; Group 4: Iopromide; Group 5: Iodephor; Group 6: Iobitridol.

Six subgroups were identified according to the type of contrast media (Iohexol as reference): Iodixanol, Iobitridol, Iopamidol, Iomeprol, Iopromide, and Iodephor. Among them, the combined effect size OR and 95% CI of Iodixanol was 1.04 (0.72, 1.51), while the values for Iobitridol were 0.97 (0.49, 1.93), Iopamidol 1.51 (0.94, 2.44), Iomeprol 3.31 (1.58, 6.91), Iopromide 3.71 (1.68, 8.18), and Iodephor 2.36 (1.36, 4.11). The details are shown in Figure 3.

### 3.4 Sensitivity analyses

For studies with I^2^ > 50% and more than two articles among the influencing factors, sensitivity analysis was conducted using the item-by-item exclusion method. After excluding individual studies one-by-one, the results indicated that the composite effect size did not change significantly before and after exclusion, suggesting that the results of the meta-analysis were relatively stable.

### 3.5 Publication bias

**Figure 4:**
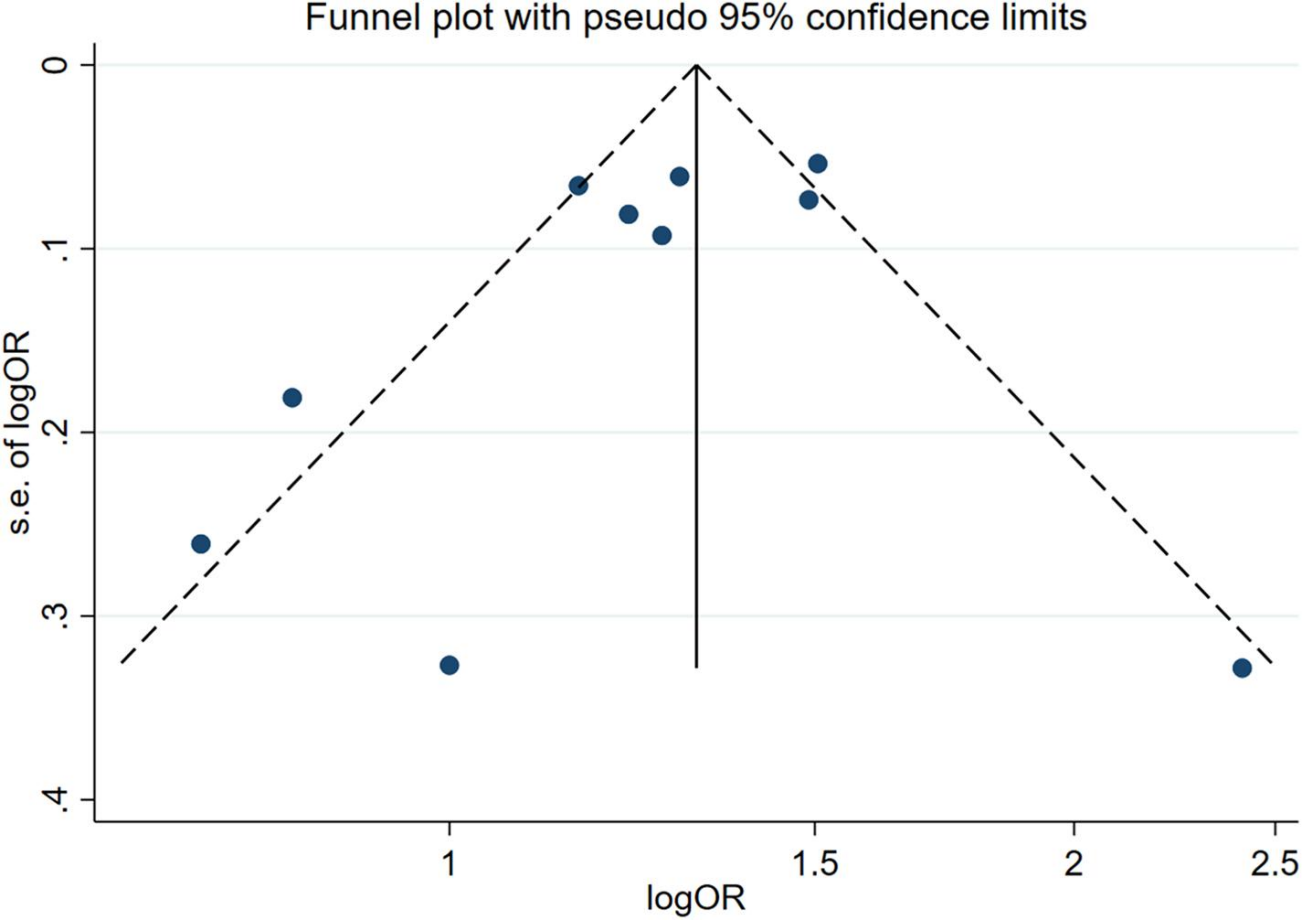
Funnel plot for assessing publication bias.

**Figure 5:**
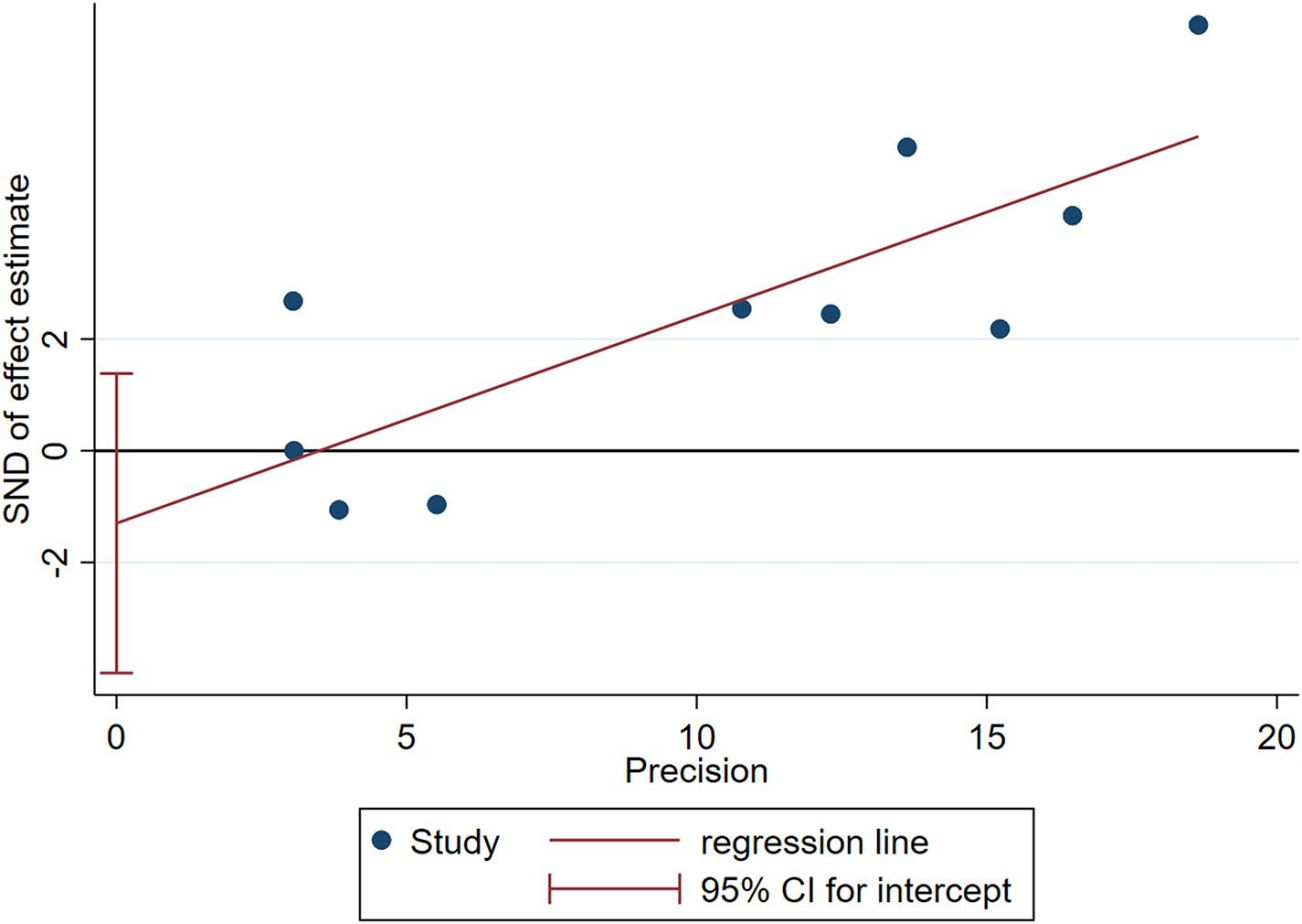
Egger's test for assessing publication bias in terms of sex.

**Figure 6:**
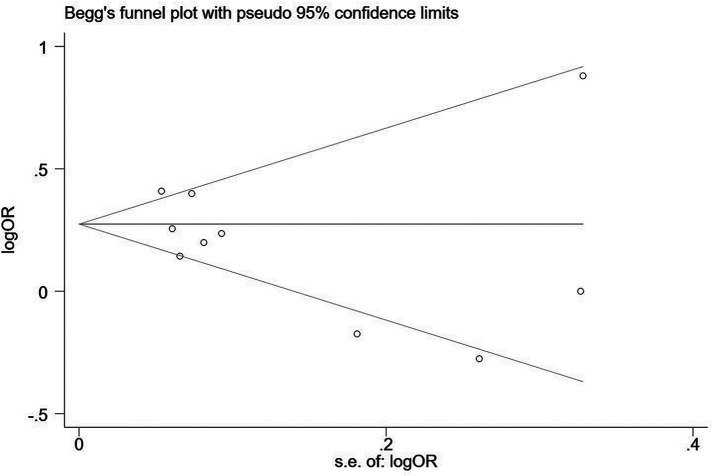
Begg's test for assessing publication bias in terms of sex.

Two influencing factors, sex and age, were described in ≥10 articles. Age, as a continuous variable, was classified in various ways across different studies, which limited direct application of a funnel plot to detect publication bias. Therefore, publication bias was only assessed for sex. The funnel plot showed good symmetry (Figure 4). Egger's test yielded a value of *P* = 0.297 and Begg's test yielded *P* = 0.592, indicating an absence of significant publication bias in the meta-analysis and enhancing the reliability of the results. Details are shown in Figures 5, 6.

## 4 Discussion

### 4.1 Socio-demographic factors

The findings indicated that female patients had a 27% increased risk of ICM-AARs compared to male patients. This result suggests that females may be more sensitive to ICM, and therefore female patients should exercise greater caution when using ICM. These findings are not consistent with the results of a meta-analysis conducted by Lee et al. (36). This discrepancy may be due to the more defined target population, broader population coverage, and larger sample size in the present study, all of which could have influenced the study results. These findings suggest that differences in sex may be relevant in assessing ICM-AARs risk and warrant further investigation in broader and more diverse populations.

Comparison of the risk of ICM-AARs among different age groups indicated that the risk was lower in the older group (> 60 years) and higher in the younger group (<35 years), relative to the middle-aged group (35‒60 years). This may be attributed to older individuals having reduced sensory perception and relatively higher tolerance (37, 38). Another explanation is that younger patients lack knowledge of the examination process, have relatively weaker psychological tolerance, and experience more anxiety during the examination, thus exacerbating the physiological reaction (20). It is recommended that healthcare professionals pay more attention to young patients, strengthen examination-related education, and alleviate the anxiety of patients.

A correlation was observed between ICM-AARs and body mass index. Higher body mass index values have been linked to stronger immune and histamine responses (39), and consequently, a higher risk of ICM -AARs. Although it was found that the correlation between body weight and ICM-AARs was not significant, this factor should not be ignored in clinical practice.

The study results indicated a higher risk of ICM-AARs in outpatients compared to inpatients, consistent with the findings of Dean et al. (40). Mild reactions in inpatients may be under-recognized due to reduced perception resulting from sedation or altered mental status (27). In contrast, outpatients may more readily perceive contrast-induced discomfort due to extended monitoring and the lack of sedation.

### 4.2 Disease-related factors

The results showed that the combined effect size OR for patients with a history of adverse reactions to ICM vs. those without a history of adverse reactions to ICM was 11.03 with a 95% CI (2.25, 53.97). A history of ICM-related adverse reactions was defined as the appearance of allergic-like responses (e.g., urticaria) or physiological reactions (e.g., edema) within one hour of ICM administration, or from one hour up to several days after ICM administration. It is hypothesized that previous adverse reactions may have triggered or augmented the immune response in patients, making them more susceptible to adverse reactions when re-exposed to ICM. Therefore, this factor should prioritized as an important risk indicator in clinical practice.

The results suggest that premedication may act as a protective factor against ICM-AARs. Currently, drugs such as antihistamines or steroids can be used prophylactically to reduce the incidence of adverse reactions before ICM injection in patients with a history of mild adverse reactions (41). However, drugs such as sterolds are not effective in preventing adverse reactions in patients with a history of moderate to severe adverse reactions to ICM (42). One study noted that in patients with a prior history of severe adverse reactions to ICM, replacement of the ICM with one having a different side chain to the original ICM helped reduce the incidence of serious adverse reactions (43). However, we acknowledge a key limitation in that the definition and application of this variable were not standardized among the different studies. Future research should aim to clearly define and standardize premedication protocols to better inform clinical risk management and preventive strategies.

The findings indicated that a history of asthma, hyperthyroldism, and other allergies were significant risk factors for ICM-AARs. Asthmatic patients may exhibit stronger allergic reactions to ICM due to airway hyperresponsiveness and specific immune responses (44). Patients with hyperthyroldism may also be at increased risk of adverse reactions due to the iodine content of ICM, potentially leading to further disturbances in thyrold function (2). Furthermore, patients with a history of allergies and previous adverse reactions to ICM may exhibit increased sensitivity to ICM, necessitating special attention to these patients when administering ICM. The present study also observed an interesting phenomenon, specifically, patients with hypertension appeared to be at a lower risk of ICM-AARs. Hypertensive patients may have a reduced risk of ICM-AARs due to the long-term use of antihypertensive medications, potentially increasing their tolerance to these agents. However, few studies have addressed these factors, and further pharmacological studies are necessary to verify these findings.

### 4.3 Iodine contrast media-related factors

The subgroup analyses of ICM types indicated that Iomeprol, Iopromide, and Iodephor were associated with a higher risk of ICM-AARs compared to Iohexol. The differences between Iodixanol, Iobitridol, Iopamidol, and Iohexol were, however, not statistically significant. A study by Terrenato et al. (45) observed a higher incidence of Iopromide-related adverse reactions compared to Iodixanol, possibly due to the hemodynamic effects of the former, which may lead to transiently increased heart rate and decreased blood pressure (46). Additionally, the American Handbook for the Use of Radiological Contrast Media (47) reported that the overall incidence of Iopromide-related reactions was 0.7%, while the incidence of acute anaphylactic-like reactions for Iohexol and Iodixanol was 0.6%. These statistics are essentially consistent with the results obtained in this study. Based on our findings, in the absence of special circumstances, it is recommended that ICMs associated with a lower risk of adverse reactions be used to ensure patient safety. With the increasing diversity of ICM currently used in clinical practice, future studies should provide more detailed reporting on key physicochemical properties, such as osmolality and iodine concentration, to enable detailed mechanistic analyses and risk stratification. Due to the different descriptors of injection flow rate (19, 22, 28) and injection dose (19, 27, 28), these parameters could not be effectively combined to analyse their effects on ICM-AARs, and more in-depth studies are needed in the future.

Of the 17 studies included in this meta-analysis, the majority were conducted in China and South Korea, which may limit the generalizability of the findings to populations in other regions. Differences in the usage patterns of contrast media, patient physiology, and genetic background between Asian and Western populations could influence the risk patterns associated with ICM-AARs. Therefore, it is suggested that studies from Europe and the USA be included in the future to enhance the universality and external validity of the conclusions.

The present study utilized the Newcastle-Ottawa Scale (NOS) and the Agency for Healthcare Research and Quality (AHRQ) checklist to assess the methodological quality of the included studies, considering a score of ≥6 as indicative of high quality. However, we acknowledge that this threshold may be relatively lenient, particularly when synthesizing data with substantial clinical heterogeneity. Although the majority of the included studies demonstrated acceptable quality scores, variations in methodological rigor may still have introduced potential bias in the pooled estimates. To address this, sensitivity analyses were performed to examine the robustness of the results in terms of study quality. Future research may benefit from the application of stricter or tiered quality criteria to enhance the interpretability and credibility of the findings of meta-analyses.

In this meta-analysis, several pooled effects were found to show extremely high statistical heterogeneity, with I^2^ values generally exceeding 90%, indicating substantial variability among the included studies. Possible sources of this heterogeneity include differences in study design and geographical variations. Although most analyses reached statistical significance, these findings should be interpreted with caution given the extent of heterogeneity.

The study has several limitations. First, all the included studies were observational in nature, which may introduce inherent biases. Second, the age group classifications were determined through discussion based on previous studies, potentially introducing selection bias. Third, in cases where the event incidence was below 10%, certain hazard ratios (HRs) (31) and risk differences (RDs) (28) were approximated as odds ratios (ORs). Although this approximation is unlikely to have substantially affected the overall statistical significance of the results, it may have reduced the interpretability of some pooled effect estimates. Moreover, moderate to high heterogeneity was observed in certain subgroup analyses, which may weaken the strength of some conclusions. While random-effects models were used to account for between-study variability, the heterogeneity may reflect differences in study design, populations, or outcome definitions. Therefore, the results should be interpreted with caution, particularly in subgroups with substantial heterogeneity. Furthermore, to ensure the methodological quality of the included studies, those with quality scores below 6 were excluded. While this approach could enhance the reliability of the pooled estimates, it may also introduce selection bias by the exclusion of potentially relevant studies with lower scores. Finally, this study did not include all possible influencing factors, and future research should continue to explore additional risk factors associated with ICM-AARs.

## 5 Conclusion

This meta-analysis identified risk factors and protective factors associated with ICM-AARs. Female sex, age <35 years, high body mass index, outpatient status, history of asthma, hyperthyroldism, history of other allergies, history of ICM-AARs, and type of ICM (Iomeprol, Iopromide, Ioversol) were found to be risk factors for ICM-AARs, whereas age >60 years, pre-injection medication, and hypertensive disorders were identified as protective factors. These findings may provide a useful reference for clinical risk assessment, particularly in settings comparable to the populations studied, though further prospective verification is needed.

## Data Availability

The original contributions presented in the study are included in the article/Supplementary Material, further inquiries can be directed to the corresponding author.
