# Supplementary material for: Predictors of acute adverse reactions to non-ionic iodinated contrast media in CT imaging: a systematic review and meta-analysis
Source: Front Radiol. 2025 Sep 19;5:1656949. doi: 10.3389/fradi.2025.1656949 (PMC12491283; doi:10.3389/fradi.2025.1656949)
Supplement: Supplementary file 1 [file Table1.docx]

**Supplementary Table S1 Search strategy**

|  | **CNKI** | Results |
| --- | --- | --- |
| #1 | SU %= '碘'+'造影剂'+'对比剂'+'碘对比剂'+'碘造影剂'+'CT对比剂' |  |
| #2 | SU %= '药物相关的副作用'+'不良反应'+'药物不良反应'+'药物不良事件'+'药物毒性'+'急性不良反应' |  |
| #3 | SU %= '影响因素'+'危险因素'+'相关因素'+'因素' |  |
| #4 | **#1 AND #2 AND #3** | 136 |

|  | **Wanfang** | Results |
| --- | --- | --- |
| #1 | （ 主题:(碘 or 造影剂 or 对比剂 or 碘对比剂 or 碘造影剂 or CT对比剂)） |  |
| #2 | （主题:(药物相关的副作用和不良反应 or 药物不良反应 or 药物不良事件 or 药物毒性 or 不良反应 or 急性不良反应) ） |  |
| #3 | （主题:(影响因素 or 危险因素 or 相关因素 or 因素)） |  |
| #4 | **#1 AND #2 AND #3** | 599 |

|  | **VIP** | Results |
| --- | --- | --- |
| #1 | (M=(碘 OR 造影剂 OR 对比剂 OR 碘对比剂 OR 碘造影剂 OR CT对比剂)) |  |
| #2 | (M=(药物相关的副作用和不良反应 OR 药物不良反应 OR 药物不良事件 OR 药物毒性 OR 不良反应 OR 急性不良反应)) |  |
| #3 | (M=(影响因素 OR 危险因素 OR 相关因素 OR 因素)) |  |
| #4 | **#1 AND #2 AND #3** | 180 |

|  | **CBM** | Results |
| --- | --- | --- |
| #1 | ( "碘"[常用字段:智能] OR "造影剂"[常用字段:智能] OR "对比剂"[常用字段:智能] OR "碘对比剂"[常用字段:智能] OR "碘造影剂"[常用字段:智能] OR "CT对比剂"[常用字段:智能]) |  |
| #2 | ( "药物相关的副作用和不良反应"[常用字段:智能] OR "药物不良反应"[常用字段:智能] OR "药物不良事件"[常用字段:智能] OR "药物毒性"[常用字段:智能] OR "不良反应"[常用字段:智能] OR "急性不良反应"[常用字段:智能]) |  |
| #3 | ( "影响因素"[常用字段:智能] OR "危险因素"[常用字段:智能] OR "相关因素"[常用字段:智能] OR "因素"[常用字段:智能]) |  |
| #4 | **#1 AND #2 AND #3** | 364 |

|  | **Pubmed** | Results |
| --- | --- | --- |
| #1 | Contrast Media"[Mesh] |  |
| #2 | (((((((((((((((((Media, Contrast[Title/Abstract]) OR (Contrast Agent[Title/Abstract])) OR (Agent, Contrast[Title/Abstract])) OR (Contrast Agents[Title/Abstract])) OR (Agents, Contrast[Title/Abstract])) OR (Contrast Materials[Title/Abstract])) OR (Materials, Contrast[Title/Abstract])) OR (Contrast Material[Title/Abstract])) OR (Material, Contrast[Title/Abstract])) OR (Radiocontrast Media[Title/Abstract])) OR (Media, Radiocontrast[Title/Abstract])) OR (Radiopaque Media[Title/Abstract])) OR (Media, Radiopaque[Title/Abstract])) OR (Radiocontrast Agents[Title/Abstract])) OR (Agents, Radiocontrast[Title/Abstract])) OR (Radiocontrast Agent[Title/Abstract])) OR (Agent, Radiocontrast[Title/Abstract])) OR (Iodinated contrast[Title/Abstract]) |  |
| #3 | #1OR#2 |  |
| #4 | Drug-Related Side Effects and Adverse Reactions"[Mesh] |  |
| #5 | (((((((((((((((((((((((((Drug-Related Side Effects[Title/Abstract]) OR (Adverse Reactions[Title/Abstract])) OR (Drug Related Side Effects[Title/Abstract] AND Adverse Reactions[Title/Abstract])) OR (Drug-Related Side Effects[Title/Abstract] AND Adverse Reaction[Title/Abstract])) OR (Drug Side Effects[Title/Abstract])) OR (Drug Side Effect[Title/Abstract])) OR (Effects, Drug Side[Title/Abstract])) OR (Side Effect, Drug[Title/Abstract])) OR (Side Effects, Drug[Title/Abstract])) OR (Adverse Drug Reaction[Title/Abstract])) OR (Adverse Drug Reactions[Title/Abstract])) OR (Drug Reaction, Adverse[Title/Abstract])) OR (Drug Reactions, Adverse[Title/Abstract])) OR (Reactions, Adverse Drug[Title/Abstract])) OR (Adverse Drug Event[Title/Abstract])) OR (Adverse Drug Events[Title/Abstract])) OR (Drug Event, Adverse[Title/Abstract])) OR (Drug Events, Adverse[Title/Abstract])) OR (Side Effects of Drugs[Title/Abstract])) OR (Drug Toxicity[Title/Abstract])) OR (Toxicity, Drug[Title/Abstract])) OR (Drug Toxicities[Title/Abstract])) OR (Toxicities, Drug[Title/Abstract])) OR (Acute reaction[Title/Abstract])) OR (adverse effects[Title/Abstract])) OR (Acute adverse reaction[Title/Abstract]) |  |
| #6 | #4 OR #5 |  |
| #7 | "Risk Factors"[Mesh] |  |
| #8 | ((((((((((((((((((((((Factor, Risk[Title/Abstract]) OR (Risk Factor[Title/Abstract])) OR (Population at Risk[Title/Abstract])) OR (Populations at Risk[Title/Abstract])) OR (Risk Scores[Title/Abstract])) OR (Risk Score[Title/Abstract])) OR (Score, Risk[Title/Abstract])) OR (Risk Factor Scores[Title/Abstract])) OR (Risk Factor Score[Title/Abstract])) OR (Score, Risk Factor[Title/Abstract])) OR (Health Correlates[Title/Abstract])) OR (Correlates, Health[Title/Abstract])) OR (Social Risk Factors[Title/Abstract])) OR (Factor, Social Risk[Title/Abstract])) OR (Factors, Social Risk[Title/Abstract])) OR (Risk Factor, Social[Title/Abstract])) OR (Risk Factors, Social[Title/Abstract])) OR (Social Risk Factor[Title/Abstract])) OR (related factors[Title/Abstract])) OR (relevant factor[Title/Abstract])) OR (influence factor[Title/Abstract])) OR (influencing factor[Title/Abstract])) OR (factor risk[Title/Abstract]) |  |
| #9 | #7 OR #8 |  |
| #10 | #3 AND #6 AND #9 | 364 |

|  | **Embase** | Results |
| --- | --- | --- |
| #1 | 'contrast medium'/exp OR 'contrast medium' |  |
| #2 | 'media, contrast':ab,ti OR 'contrast agent':ab,ti OR 'agent, contrast':ab,ti OR 'contrast agents':ab,ti OR 'agents, contrast':ab,ti OR 'contrast materials':ab,ti OR 'materials, contrast':ab,ti OR 'contrast material':ab,ti OR 'material, contrast':ab,ti OR 'radiocontrast media':ab,ti OR 'media, radiocontrast':ab,ti OR 'radiopaque media':ab,ti OR 'media, radiopaque':ab,ti OR 'radiocontrast agents':ab,ti OR 'agents, radiocontrast':ab,ti OR 'radiocontrast agent':ab,ti OR 'agent, radiocontrast':ab,ti OR 'iodinated contrast':ab,ti |  |
| #3 | #1 OR #2 |  |
| #4 | 'adverse drug reaction'/exp OR 'adverse drug reaction' |  |
| #5 | 'drug-related side effects':ab,ti OR 'adverse reactions':ab,ti OR 'drug related side effects[title/abstract] and adverse reactions':ab,ti OR 'drug-related side effects[title/abstract] and adverse reaction':ab,ti OR 'drug side effects':ab,ti OR 'drug side effect':ab,ti OR 'effects, drug side':ab,ti OR 'side effect, drug':ab,ti OR 'side effects, drug':ab,ti OR 'adverse drug reaction':ab,ti OR 'adverse drug reactions':ab,ti OR 'drug reaction, adverse':ab,ti OR 'drug reactions, adverse':ab,ti OR 'reactions, adverse drug':ab,ti OR 'adverse drug event':ab,ti OR 'adverse drug events':ab,ti OR 'drug event, adverse':ab,ti OR 'drug events, adverse':ab,ti OR 'side effects of drugs':ab,ti OR 'drug toxicity':ab,ti OR 'toxicity, drug':ab,ti OR 'drug toxicities':ab,ti OR 'toxicities, drug':ab,ti OR 'acute reaction':ab,ti OR 'adverse effects':ab,ti OR 'acute adverse reaction':ab,ti |  |
| #6 | #4 OR #5 |  |
| #7 | 'risk factor'/exp OR 'risk factor' |  |
| #8 | 'factor, risk':ab,ti OR 'risk factor':ab,ti OR 'population at risk':ab,ti OR 'populations at risk':ab,ti OR 'risk scores':ab,ti OR 'risk score':ab,ti OR 'score, risk':ab,ti OR 'risk factor scores':ab,ti OR 'risk factor score':ab,ti OR 'score, risk factor':ab,ti OR 'health correlates':ab,ti OR 'correlates, health':ab,ti OR 'social risk factors':ab,ti OR 'factor, social risk':ab,ti OR 'factors, social risk':ab,ti OR 'risk factor, social':ab,ti OR 'risk factors, social':ab,ti OR 'social risk factor':ab,ti OR 'related factors':ab,ti OR 'relevant factor':ab,ti OR 'influence factor':ab,ti OR 'influencing factor':ab,ti OR 'factor risk':ab,ti |  |
| #9 | #7 OR #8 |  |
| #10 | #3 AND #6 AND #9 | 3307 |

|  | **Web of science** | Results |
| --- | --- | --- |
| #1 | contrast media (Topic) or Media, Contrast (Topic) or Contrast Agent (Topic) or Agent, Contrast (Topic) or Contrast Agents (Topic) or Agents, Contrast (Topic) or Contrast Materials (Topic) or Materials, Contrast (Topic) or Contrast Material (Topic) or Material, Contrast (Topic) or Radiocontrast Media (Topic) or Media, Radiocontrast (Topic) or Radiopaque Media (Topic) or Media, Radiopaque (Topic) or Radiocontrast Agents (Topic) or Agents, Radiocontrast (Topic) or Radiocontrast Agent (Topic) or Agent, Radiocontrast (Topic) or Iodinated contrast (Topic) |  |
| #2 | Drug-Related Side Effects and Adverse Reactions (Topic) or Drug-Related Side Effects (Topic) or Adverse Reactions (Topic) or Drug Related Side Effects and Adverse Reactions (Topic) or Drug-Related Side Effects and Adverse Reaction (Topic) or Drug Side Effects (Topic) or Drug Side Effect (Topic) or Effects, Drug Side (Topic) or Side Effect, Drug (Topic) or Side Effects, Drug (Topic) or Adverse Drug Reaction (Topic) or Adverse Drug Reactions (Topic) or Drug Reaction, Adverse (Topic) or Drug Reactions, Adverse (Topic) or Reactions, Adverse Drug (Topic) or Adverse Drug Event (Topic) or Adverse Drug Events (Topic) or Drug Event, Adverse (Topic) or Drug Events, Adverse (Topic) or Side Effects of Drugs (Topic) or Drug Toxicity (Topic) or Toxicity, Drug (Topic) or Drug Toxicities (Topic) or Toxicities, Drug (Topic) or Acute reaction (Topic) or adverse effects (Topic) or Acute adverse reaction (Topic) |  |
| #3 | Risk Factors (Topic) or Factor, Risk (Topic) or Risk Factor (Topic) or Population at Risk (Topic) or Populations at Risk (Topic) or Risk Scores (Topic) or Risk Score (Topic) or Score, Risk (Topic) or Risk Factor Scores (Topic) or Risk Factor Score (Topic) or Score, Risk Factor (Topic) or Health Correlates (Topic) or Correlates, Health (Topic) or Social Risk Factors (Topic) or Factor, Social Risk (Topic) or Factors, Social Risk (Topic) or Risk Factor, Social (Topic) or Risk Factors, Social (Topic) or Social Risk Factor (Topic) or related factors (Topic) or relevant factor (Topic) or influence factor (Topic) or influencing factor (Topic) or factor risk (Topic) |  |
| #4 | **#1 AND #2 AND #3** | 686 |

|  | **Cochrane Library** | Results |
| --- | --- | --- |
| #1 | MeSH descriptor: [Contrast Media] explode all trees |  |
| #2 | (Media, Contrast):ti,ab,kw OR (Contrast Agent):ti,ab,kw OR (Agent, Contrast):ti,ab,kw OR (Contrast Agents):ti,ab,kw OR (Agents, Contrast):ti,ab,kw OR (Contrast Materials):ti,ab,kw OR (Materials, Contrast):ti,ab,kw OR (Contrast Material):ti,ab,kw OR (Material, Contrast):ti,ab,kw OR (Radiocontrast Media):ti,ab,kw OR (Media, Radiocontrast):ti,ab,kw OR (Radiopaque Media):ti,ab,kw OR (Media, Radiopaque):ti,ab,kw OR (Radiocontrast Agents):ti,ab,kw OR (Agents, Radiocontrast):ti,ab,kw OR (Radiocontrast Agent):ti,ab,kw OR (Agent, Radiocontrast):ti,ab,kw OR (Iodinated contrast):ti,ab,kw |  |
| #3 | #1 OR #2 |  |
| #4 | MeSH descriptor: [Drug-Related Side Effects and Adverse Reactions] explode all trees |  |
| #5 | (Drug-Related Side Effects):ti,ab,kw OR (Adverse Reactions):ti,ab,kw OR (Drug Related Side Effects and Adverse Reactions):ti,ab,kw OR (Drug-Related Side Effects and Adverse Reaction):ti,ab,kw OR (Drug Side Effects):ti,ab,kw OR (Drug Side Effect):ti,ab,kw OR (Effects, Drug Side):ti,ab,kw OR (Side Effect, Drug):ti,ab,kw OR (Side Effects, Drug):ti,ab,kw OR (Adverse Drug Reaction):ti,ab,kw OR (Adverse Drug Reactions):ti,ab,kw OR (Drug Reaction, Adverse):ti,ab,kw OR (Drug Reactions, Adverse):ti,ab,kw OR (Reactions, Adverse Drug):ti,ab,kw OR (Adverse Drug Event):ti,ab,kw OR (Adverse Drug Events):ti,ab,kw OR (Drug Event, Adverse):ti,ab,kw OR (Drug Events, Adverse):ti,ab,kw OR (Side Effects of Drugs):ti,ab,kw OR (Drug Toxicity):ti,ab,kw OR (Toxicity, Drug):ti,ab,kw OR (Drug Toxicities):ti,ab,kw OR (Toxicities, Drug):ti,ab,kw OR (Acute reaction):ti,ab,kw OR (adverse effects):ti,ab,kw OR (Acute adverse reaction):ti,ab,kw |  |
| #6 | #4 OR #5 |  |
| #7 | MeSH descriptor: [Risk Factors] explode all trees |  |
| #8 | (Factor, Risk):ti,ab,kw OR (Risk Factor):ti,ab,kw OR (Population at Risk):ti,ab,kw OR (Populations at Risk):ti,ab,kw OR (Risk Scores):ti,ab,kw OR (Risk Score):ti,ab,kw OR (Score, Risk):ti,ab,kw OR (Risk Factor Scores):ti,ab,kw OR (Risk Factor Score):ti,ab,kw OR (Score, Risk Factor):ti,ab,kw OR (Health Correlates):ti,ab,kw OR (Correlates, Health):ti,ab,kw OR (Social Risk Factors):ti,ab,kw OR (Factor, Social Risk):ti,ab,kw OR (Factors, Social Risk):ti,ab,kw OR (Risk Factor, Social):ti,ab,kw OR (Risk Factors, Social):ti,ab,kw OR (Social Risk Factor):ti,ab,kw OR (related factors):ti,ab,kw OR (relevant factor):ti,ab,kw OR (influence factor):ti,ab,kw OR (influencing factor):ti,ab,kw OR (factor risk):ti,ab,kw |  |
| #9 | #7 OR #8 |  |
| #10 | #3 AND #6 AND #9 | 930 |
