# Supplementary material for: Predictors of acute adverse reactions to non-ionic iodinated contrast media in CT imaging: a systematic review and meta-analysis
Source: Front Radiol. 2025 Sep 19;5:1656949. doi: 10.3389/fradi.2025.1656949 (PMC12491283; doi:10.3389/fradi.2025.1656949)
Supplement: Supplementary file 2 [file Table2.docx]

### Table S2. Quality assessment score.[ Cohort Studies：NOS]

| **Author; year** | **Selection**  **1- Representativeness of the exposed cohort** | **2- Selection of the non exposed cohort** | **3-Ascertainment of exposure** | **4- Demonstration that outcome of interest was not present at start of study** | **Comparability**  **1- Comparability of cohorts on the basis of the design or analysis** | **Outcome**  **1- Assessment of outcome** | **2-**  **Was follow-up long enough for outcomes to occur** | **3-Adequacy of follow up of cohorts** | **Quality**  **score** |
| --- | --- | --- | --- | --- | --- | --- | --- | --- | --- |
| **Xu et al，2023** | 1 | 1 | 1 | 0 | 2 | 1 | 0 | 1 | 7 |
| **Liu et al，2023** | 1 | 1 | 1 | 1 | 1 | 1 | 0 | 1 | 7 |
| **Park et al，2019** | 1 | 1 | 1 | 1 | 2 | 1 | 0 | 1 | 8 |
| **Yang et al，2015** | 1 | 1 | 1 | 1 | 1 | 1 | 1 | 0 | 7 |

### Quality assessment score. [ Case-Control Studies:NOS]

| **Author; year** | **Selection**  **1-Is the case definition adequate?** | **2-Representativeness of the cases** | **3-Selection of Controls** | **4- Definition of Controls** | **Comparability**  **1- Comparability of cases and controls on the basis of the design or analysis** | **Outcome**  **1-Ascertainment of exposure** | **2-**  **Same method of ascertainment for cases and controls** | **3-**  **Non-Response rate** | **Quality**  **score** |
| --- | --- | --- | --- | --- | --- | --- | --- | --- | --- |
| **Qiu et al，2023** | 1 | 1 | 1 | 1 | 1 | 1 | 1 | 1 | 8 |
| **Gao et al，2023** | 1 | 1 | 1 | 1 | 1 | 1 | 1 | 1 | 8 |
| **Ding et al，2023** | 1 | 1 | 1 | 1 | 1 | 1 | 1 | 1 | 8 |
| **Lin et al，2022** | 1 | 1 | 1 | 1 | 1 | 1 | 1 | 0 | 7 |
| **Gan et al，2020** | 1 | 1 | 1 | 1 | 1 | 1 | 1 | 1 | 8 |
| **Zeng et al，2024** | 1 | 1 | 1 | 1 | 1 | 1 | 1 | 1 | 8 |
| **McDonald et al，2023** | 1 | 1 | 1 | 1 | 1 | 1 | 1 | 0 | 7 |
| **Fukushima et al，2023** | 1 | 1 | 1 | 0 | 1 | 1 | 1 | 1 | 7 |
| **Kim et al，2017** | 1 | 1 | 1 | 1 | 1 | 1 | 1 | 1 | 8 |
| **Ho et al，2012** | 1 | 1 | 1 | 1 | 1 | 1 | 1 | 0 | 7 |

### Quality assessment score.[Cross-Sectional Studies:AHRQ]

| **Author; year** | **1-Define the source of information (survey, record review).** | **2-List inclusion and exclusion criteria for exposed and unexposed subjects (cases and controls) or refer to previous publications.** | **3-Indicate time period used for identifying patients.** | **4-Indicate whether or not subjects were consecutive if not population-based.** | **5-Indicate if evaluators of subjective components of study were masked to other aspects of the participants.** | **6-Describe any assessments undertaken for quality assurance purposes (e.g., test/retest of primary outcome measurements).** | **7-Explain any patient exclusions from analysis.** | **8-Describe how confounding was assessed and/or controlled.** | **9-If applicable, explain how missing data were handled in the analysis.** | **10-Summarize patient response rates and completeness of data collection.** | **11-Clarify what follow-up, if any, was expected and the percentage of patients for which incomplete data or follow-up was obtained.** | **Quality**  **score** |
| --- | --- | --- | --- | --- | --- | --- | --- | --- | --- | --- | --- | --- |
| **Yang et al，** | 1 | 1 | 1 | 1 | 1 | 1 | 0 | 0 | 0 | 1 | 0 | 7 |
| **Lee et al，** | 1 | 0 | 1 | 1 | 1 | 1 | 0 | 1 | 0 | 0 | 0 | 6 |
| **Cha et al，** | 1 | 0 | 1 | 1 | 1 | 1 | 0 | 1 | 0 | 0 | 0 | 6 |
